# Are additional screws required for press-fit fixation of cementless acetabular cups? A systematic review and meta-analysis

**DOI:** 10.1186/s10195-022-00629-8

**Published:** 2022-02-10

**Authors:** Shenghui Ni, Peng Luo, Lei Guo, Tianlong Jiang

**Affiliations:** 1 Department of Orthopedic Surgery, First Affiliated Hospital, China Medical University, Shenyang, 110001 Liaoning China; 2 Department of Orthopedic Surgery, Fourth Affiliated Hospital, China Medical University, Shenyang, 110001 Liaoning China

**Keywords:** Total hip arthroplasty, Cementless acetabular cup, Screw

## Abstract

**Background:**

Press-fit cementless acetabular cup is widely used in total hip arthroplasty (THA). However, the use of additional screws for the acetabular cup has been extensively debated. The purpose of this review is to compare the stability, revision rate, wear rate, and clinical scores of cementless acetabular cups with and without screws in THA.

**Materials and Methods:**

Comprehensive literature searches of the following databases were performed: Cochrane Library, Pubmed, Web of Science, OVID, Elsevier ClinicalKey, Clinicaltrials.gov, and EMBASE. We searched for trials that compared cementless acetabular cups with screws or without screws, and were published in the English language. We evaluated the stability of the prosthesis by osteolysis and migration. The clinical scores included Harris hip scores (HHS) and pain scores.

**Results:**

Nineteen articles involving 4046 THAs met the inclusion criteria. Our analysis revealed that additional screws did not increase the stability of acetabular cups, and there was no statistical significance between the groups with and without screws in osteolysis and clinically relevant migration. Revision rates showed no significant difference between the groups with and without screws. There was no difference in wear between the two groups. Our analysis showed no difference in pain scores and HHS between groups.

**Conclusion:**

Press-fit without screws could achieve sufficient acetabular cup stability. Acetabular cups without screws showed no difference from acetabular cups with screws in many outcomes. Additional screws are not required for cementless acetabular cups.

*Level of evidence:* Level III.

## Introduction

Total hip arthroplasty (THA) is an effective and commonly used treatment for various pathological hip conditions. The cementless acetabular cup, which is fixed by press-fit, is widely used in THAs [1]. Most cementless acetabular cups have several screw holes for optional screw fixation. Some surgeons have suggested that adding screws can increase the stability of the acetabular cup [2]. It has been found that granulomas can reach around the screw through the screw hole, thus causing osteolysis [1]. Another radiographic follow-up of the acetabular components with screws revealed osteolysis around the screws in 17% of cases [3]. These studies suggested that osteolysis was closely related to screw holes and screws. Additionally, fixation with screws was associated with a risk of increased surgical time [4, 5]. So far, there is no definitive guide on whether to add screws on the cementless acetabular cup, and the screw addition is widely debated.

In response to this controversial clinical issue, a meta-analysis was conducted by Ni et al. in 2013 [6]. Their study revealed no significant differences in osteolysis, prosthesis loosening, or revision rates with or without the addition of screws. This was the first evidence-based study in this field and after being published, this matter received wide attention from peers and more relevant clinical studies were published [4, 7–11]. Nonetheless, the aforementioned study had some limitations. For example, it only included five articles and only three high-quality randomized controlled trials (RCTs). Hence, we have updated the previous meta-analysis by retrieving more databases and adding the latest clinical studies. The purpose of this review is to compare the stability, revision rate, wear rate, and clinical scores of cementless acetabular cups with and without screws in THA.

## Materials and methods

We conducted a systematic review according to the Preferred Reporting Items for Systematic Reviews and Meta-Analyses guidelines [12].

### Searches

A comprehensive literature search was performed using the Cochrane Library, Pubmed, Web of Science, OVID, Elsevier ClinicalKey, Clinicaltrials.gov, and EMBASE databases. The last search was run on 1 March 2021. We used the following search strategy terms: (“total hip arthroplasty”or “THA” or “total hip replacement” or “THR”) and “screw” and (“press-fit” or “press fit” or “cementless” or “uncemented” or “noncemented”) and (“osteolysis” or “migration” or “translation” or “rotation” or “revision” or “wear” or “Harris Hip score” or “pain score”). We searched only for articles published in English.

### Study inclusion and exclusion criteria

We selected all RCTs or cohort studies comparing the primary fixation of acetabular cups with or without screws during THAs. The inclusion criteria were as follows: (1) articles in English, (2) primary THA, (3) cementless press-fit acetabular cup, and (4) RCTs or cohort studies. The exclusion criteria were as follows: (1) articles not in English, (2) revision THA, (3) cement acetabular cup, (4) not clinical control studies, and (5) lack of outcomes mentioned above.

### Study quality assessment

The quality of each relevant RCT was assessed using the Jadad scale [13], where studies scoring 3–5 were designated as having a low risk of bias, and 0–2 were designated as having a high risk of bias. Also, the quality of cohort studies was assessed using the Newcastle–Ottawa scale (NOS) [14], where studies scoring 5–8 were designated as having low risk of bias, 3–4 as moderate, and 0–2 as high.

### Data extraction strategy

Two authors independently identified and evaluated the included studies. A third independent author resolved any disagreements. The data from the included articles were independently extracted by two authors, and differences were discussed with a third independent author.

### Data synthesis and presentation

Data included the measured outcomes and general characteristics of each study. For all eligible articles, the following data were extracted from original publications: study design, year of publication, number of THAs, follow–up time, operative approach, the material of acetabular component, osteolysis, migration, translation, rotation, revision, HHS, wear, and pain scores. Migration was defined as clinically relevant translation and rotation [15]. Translation and rotation of the acetabular component in each axis were measured using radiostereometric analysis (RSA). RSA is a three dimension (3D) digital radiograph technology with high accuracy [16]. The technique involves premarking the acetabular bone and components with tantalum bead implantation, taking x-ray radiographs from different angles during each examination, and using RSA software to measure and analyze the markers for each examination to calculate the relative motion of the acetabular components [9, 11, 15–19]. Translations were analyzed as medial/lateral translation (*x*-axis), proximal/distal translation (*y*-axis), anterior/posterior translation (*z*-axis), and total translation (3d). Rotations were analyzed as transverse axis rotation (*x*-axis), longitudinal axis rotation (*y*-axis), and sagittal axis rotation (*z*-axis). If the data were not directly reported, we estimated the mean and standard deviation from the median, interquartile ranges, standard errors, confidence intervals, *t* values, and *P* values [20–22]. If possible, we obtained the necessary data by contacting the authors.

We initially followed the “ITT analysis using imputation” in accordance with the Cochrane handbook for systematic reviews of interventions [20]. The statistical analysis for dichotomized outcomes was performed using the risk difference (RD) and 95% confidence interval (CI). The Mantel–Haenszel method and random-effects model were used to combine the RDs. We calculated the dichotomized outcomes using the available *P* values; a *P* < 0.05 was considered statistically significant. The mean difference (MD) and 95% CI were used for the statistical analysis of continuous variables. The inverse-variance method and random-effects model were used to combine the MDs. When a *P*-value was reported as < 0.05, the point estimate of the MD was considered statistically significant. The *I*^2^ statistic was applied to estimate the heterogeneity between trials. A *P*-value < 0.1 or *Ι*^2^ > 50% indicated significant heterogeneity.

Publication bias has long been recognized as a problem. In this review, we used a “funnel plot” to investigate the publication bias and identify signs of asymmetry.

We used the “available case analysis” in accordance with the Cochrane handbook for systematic reviews of interventions for sensitivity analysis [20]. We also performed sensitivity analyses for dichotomized outcomes using the risk ratio (RR) as an effective measure. Subgroup analyses were also performed to separately consider the randomized and nonrandomized studies. We performed a subgroup analysis for wear rate based on bearing surfaces materials. We also carried out subgroup analyses among studies involving translation and rotation in different axis and involving HHS and pain scores at different follow-up periods.

All the data collection, data extraction, and statistical analyses were conducted using Review Manager Version 5.3 from the Cochrane Collaboration.

## Results

### Review statistics

**Fig. 1:**
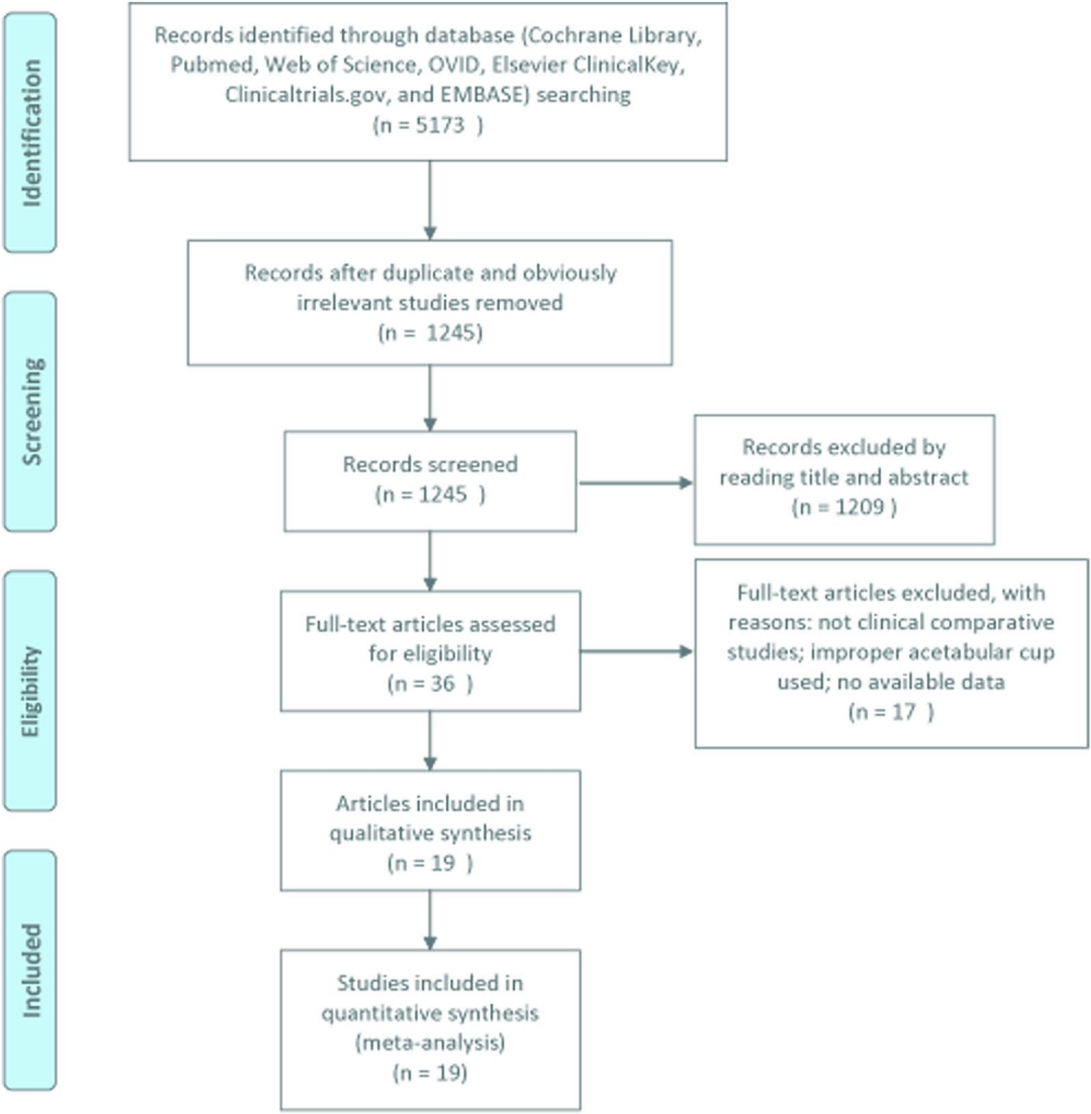
A flow chart of the study selection method

A total of 5173 publications were initially identified (Fig. 1). After a preliminary review, 3928 studies were excluded because of overlapping records or obviously irrelevant studies; 1209 articles were excluded because they were case reports, reviews, were not human studies, or were in a language other than English; and 17 articles were excluded because there was no comparison, the improper acetabular cup was used, or there was no available data. Therefore, a total of 19 articles [4, 7–11, 15, 17–19, 23–31] with 21 studies comparing the cementless press-fit acetabular cup with and without screws were included.

### Study characteristics and quality assessment

Out of the 19 included articles, 8 were RCTs and 11 were cohort studies. The characteristics of the 19 included articles are listed in Table 1. These studies had been published between 2000 and 2020 and comprised 3969 THAs. Thirteen studies with 3130 THAs reported osteolysis, and the subgroup analysis was performed by study design involving RCTs (295 THAs) and cohort studies (2835 THAs). Ten studies with 1579 THAs reported migration, and the subgroup analysis was performed by study design involving RCTs (157 THAs) and cohort studies (1422 THAs). Six studies with 976 THAs reported translation, and the subgroup analysis was performed by different axis involving the *x*-axis (280 THAs), *y*-axis (280 THAs), *z*-axis (280 THAs), and total translation/3d (136 THAs). Four studies with 609 THAs reported rotation, and the subgroup analysis was performed by different axis involving the *x*-axis (203 THAs), *y*-axis (203 THAs), and *z*-axis (203 THAs). Eleven studies with 3097 THAs reported revision, and the subgroup analysis was performed by study design involving RCTs (188 THAs) and cohort studies (2906 THAs). Four studies with 250 THAs reported pain scores, and the subgroup analysis was performed by follow-up time involving ≥ 10 years (34 THAs), 5 years ≤ and < 10 years (43 THAs), and < 5 years (173 THAs). Seven studies with 445 THAs reported the Harris Hip score, and the subgroup analysis was performed by follow-up time involving ≥ 10 years (34 THAs), 5 years ≤ and < 10 years (201 THAs), and < 5 years (210 THAs). The quality of each RCT and cohort study was assessed by the Jadad scale and NOS, respectively. The Jadad scores of the RCTs ranged from 2 to 6 with a mean of 4.5, and the NOS scores of the cohort studies ranged from 6 to 8 with a mean of 7.2 (Table 1).

**Table 1:** Characteristics and quality of the included studies

Author	Study design	ThAs (*n*)	Follow-up (year)	Operative approach	Bearing surfaces material		Outcomes	Jadad score	NOS
					Liner	Head			
Blakeney et al. [23]	RCT	56	5	Posterior	PE	Cobalt-chrome	Osteolysis; migration; revision	5	–
Gallen et al. [7]	RCT	32	10	Posterior	PE	Cobalt-chrome	Osteolysis	5	–
Garcia-Cimbrelo et al. [24]	Cohort study	319	4.7 (3–8)	Posterior	Ceramic	Ceramic	Revision	–	7
García-Rey et al. [8]	Cohort study	791	9.6 (5–15)	Posterolateral	Ceramic; PE	Ceramic; Metal	Osteolysis; migration; revision	–	8
Howie et al. [17]	RCT	66	2	Posterior	PE	Cobalt-chrome	Osteolysis; translation; Rotation; HHS	5	–
Iorio et al. [25]	Cohort study	775	2–10	NA	PE	NA	Osteolysis; migration; revision	–	8
Merican et al. [26]	Cohort study	115	2.8 (1.3–4.3)	Modified anterior	Metal	Metal	Migration; revision	–	7
Minten et al. [9]	RCT	36	6.5	Posterolateral	PE	Ceramic	Translation; rotation; HHS	5	–
Natera et al. [10]	Cohort study	749	14.2 (8.9–16.7)	Anterior; lateral; anterolateral; posterolateral	Ceramic; PE	Ceramic; Metal	Osteolysis; revision	–	7
Otten et al. [11]	RCT	34	14	Posterolateral	PE	Ceramic	Osteolysis; translation; revision; HSS	5	–
Pakvis et al. [15]	RCT	37	2	Posterolateral	PE	Ceramic	Migration; translation; rotation; revision; HHS	6	–
Pepe et al. [4]	Cohort study	30	1	Posterior	Ceramic; PE	Ceramic; Cobalt-chrome	Osteolysis; migration; revision; HHS	–	7
Röhrl et al. [18]	RCT	43	5	Posterior	PE	Cobalt-chrome; Ceramic	Osteolysis; translation; HHS	4	–
Roth et al. [27]	Cohort study	211	5	Transgluteal surgical	NA	NA	Migration	–	7
Taniguchi et al. [28]	Cohort study	209	7–10	Posterolateral	PE	Cobalt-chrome	Osteolysis; migration	–	8
Thanner et al. [19]	RCT	63	2	Modified Hardinge	PE	Ceramic; Cobalt-chrome	Osteolysis; migration; translation; rotation; revision; pain scores; HHS	2	–
Von Schewelov et al. [29]	Cohort study	154	6(0.5–12)	Lateral	PE	Cobalt-chrome	Osteolysis	–	7
Weber et al. [30]	Cohort study	127	10.6(6–13)	NA	PE	Ceramic; Metal	Osteolysis; revision	–	6
Yalcin et al. [31]	Cohort study	122	5.2 (2.7–6.3)	Posterolateral	PE; Cobalt-chrome	Cobalt-chrome	Migration; HHS	–	7

*NA* not available, *PE* polyethylene, *HHS* Harris hip scores

### Stability of acetabular cup

**Fig. 2:**
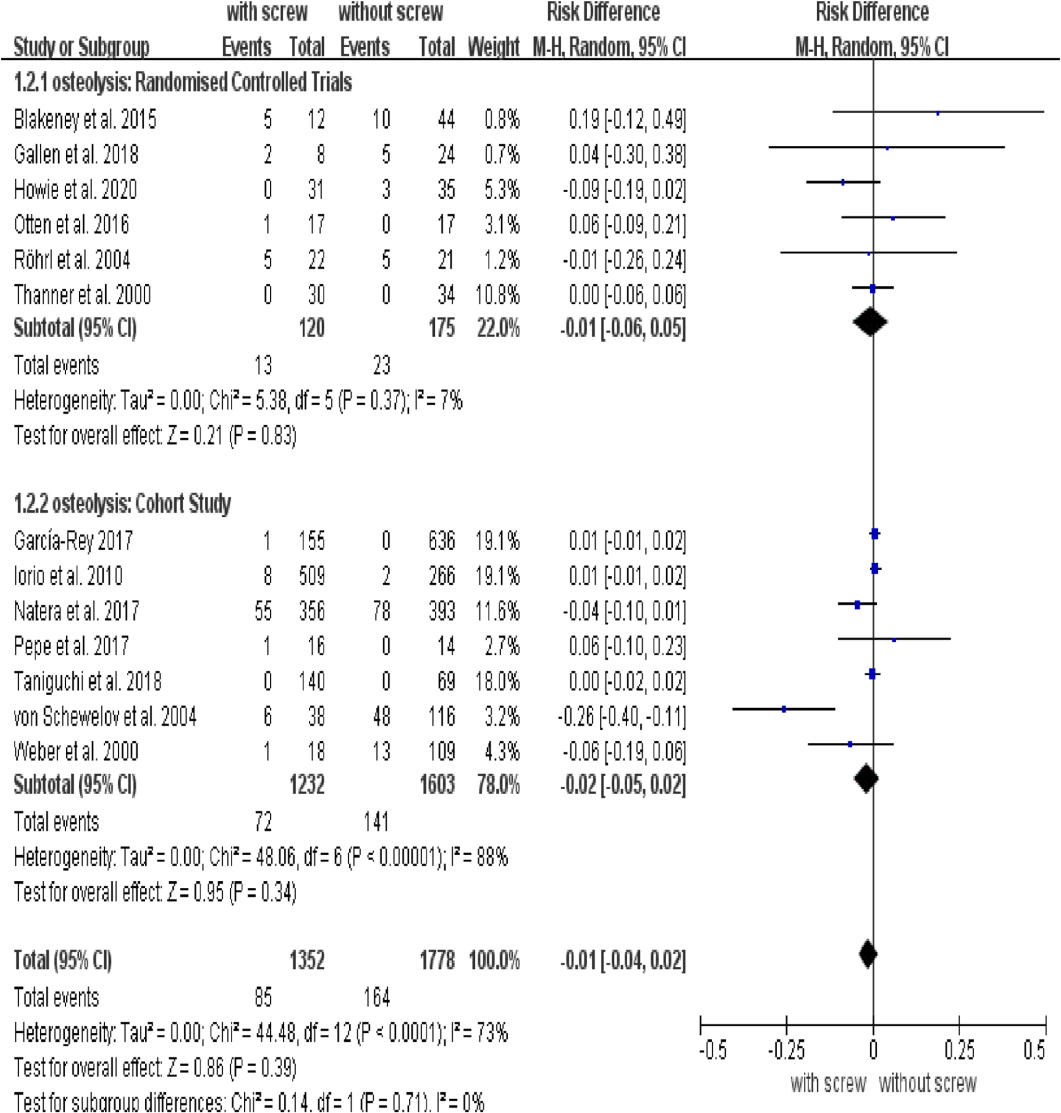
Forest plot of combined and subgroup analysis for the osteolysis of acetabular cup

**Fig. 3:**
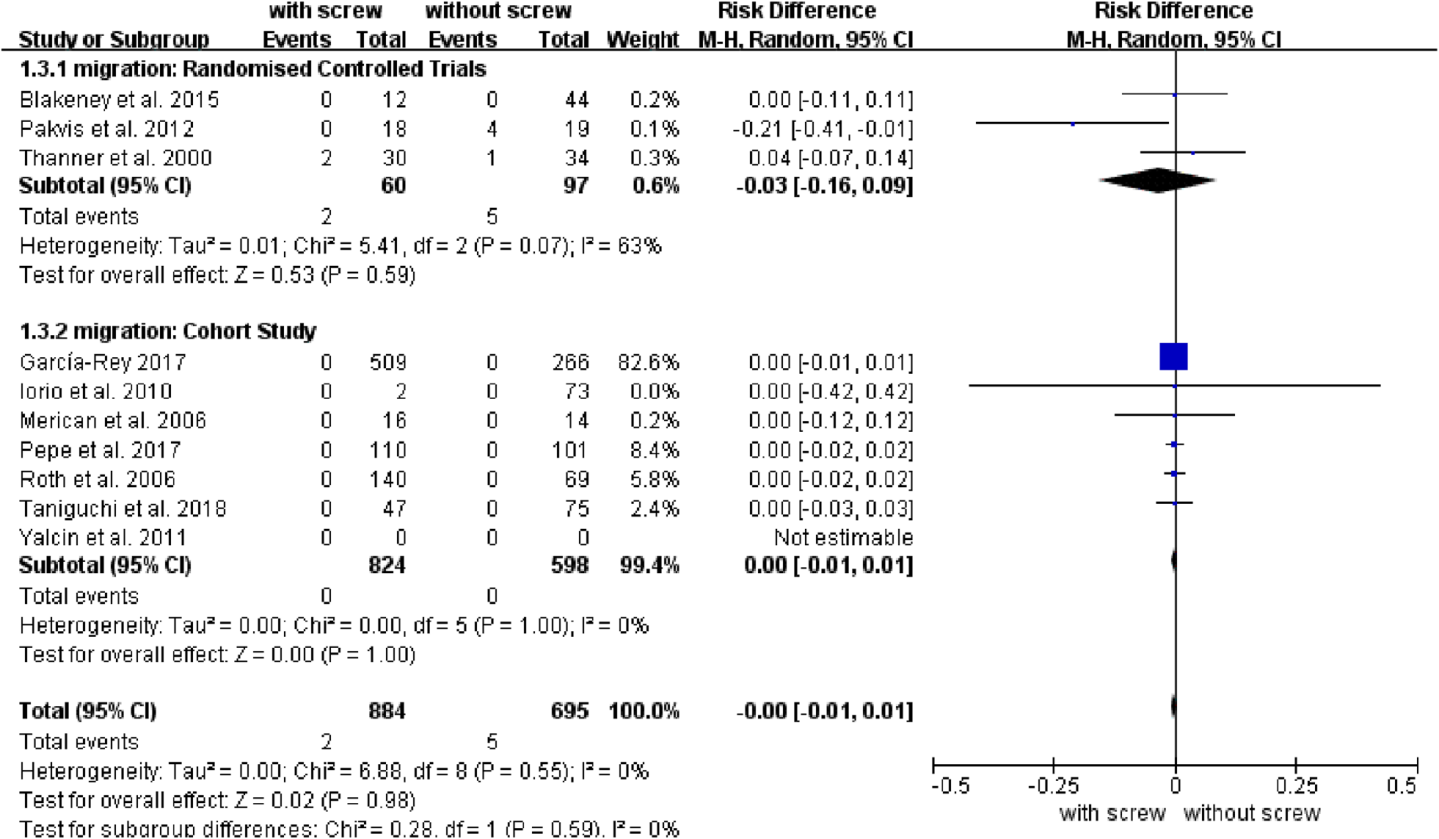
Forest plot of combined and subgroup analysis for the migration of acetabular cup

Our analysis revealed no differences in stability (osteolysis, migration, translation, and rotation) of the acetabular cup between groups. There were no significant differences in the rate of osteolysis between groups with and without screws (95% CI:  − 0.04 to 0.02; *P* = 0.39; RD = − 0.01) (Fig. 2). The RD of migration was 0, and the difference was not statistically significant (95% CI: − 0.01 to 0.01; *P* = 0.98) (Fig. 3). The MD of translation was − 0.05, and the difference was not statistically significant (95% CI: − 0.11 to 0.01; *P* = 0.11) ( Table 2). Also, no difference was observed in rotation between groups with and without screws; the MD of rotation was − 0.05 (95% CI: –0.11 to 0.01; *P* = 0.11)(Table 2). Subgroup analysis for rotation of acetabular cup showed that the rotation degree at the *x*-axis (95% CI: − 1.22 to − 0.15; *P* = 0.01; MD = − 0.68) and *z*-axis (95% CI: − 0.41 to − 0.03; *P* = 0.02; MD = − 0.22) was greater in the group without screws, and there was no difference between groups in the rotation degree at the *y*-axis (95% CI: − 2.13 to 1.93; *P* = 0.92; MD = − 0.10) (Table 2). There were also no conflicts between subgroups in osteolysis, migration, or translation (Table 2).

**Table 2:** The analysis of translation and rotation by ITT analysis using imputation

Outcomes and subgroup analysis	Studies (*n*)	THAs (*n*)	Effective measures	MD (95% CI)	*P*-value	Heterogeneity	
						*I*^2^ (%)	*P*_*h*_
Translation	21	976	MD	− 0.05(− 0.11, 0.01)	0.11	53	0.002
*X*: Medial/lateral	6	280	MD	− 0.05 (− 0.11, 0.01)	0.11	0	0.50
*Y*: Proximal/distal	6	280	MD	− 0.11 (− 0.22, 0.01)	0.07	62	0.02
*Z*: Anterior/posterior	6	280	MD	0.04 (− 0.15, 0.23)	0.70	63	0.02
Total translation/3d	3	136	MD	− 0.01 (− 0.23, 0.20)	0.28	20	0.28
Rotation	12	609	MD	− 0.31 (− 0.83, 0.21)	0.24	88	< 0.00001
*X*: Transverse axis	4	203	MD	− 0.68 (− 1.22, − 0.15)	0.01	57	0.07
*Y*: Longitudinal axis	4	203	MD	− 0.10 (− 2.13, 1.93)	0.92	96	< 0.00001
*Z*: Sagittal axis	4	203	MD	− 0.22 (− 0.41, − 0.03)	0.02	0	0.81

### Revision rate of the acetabular cup

**Fig. 4:**
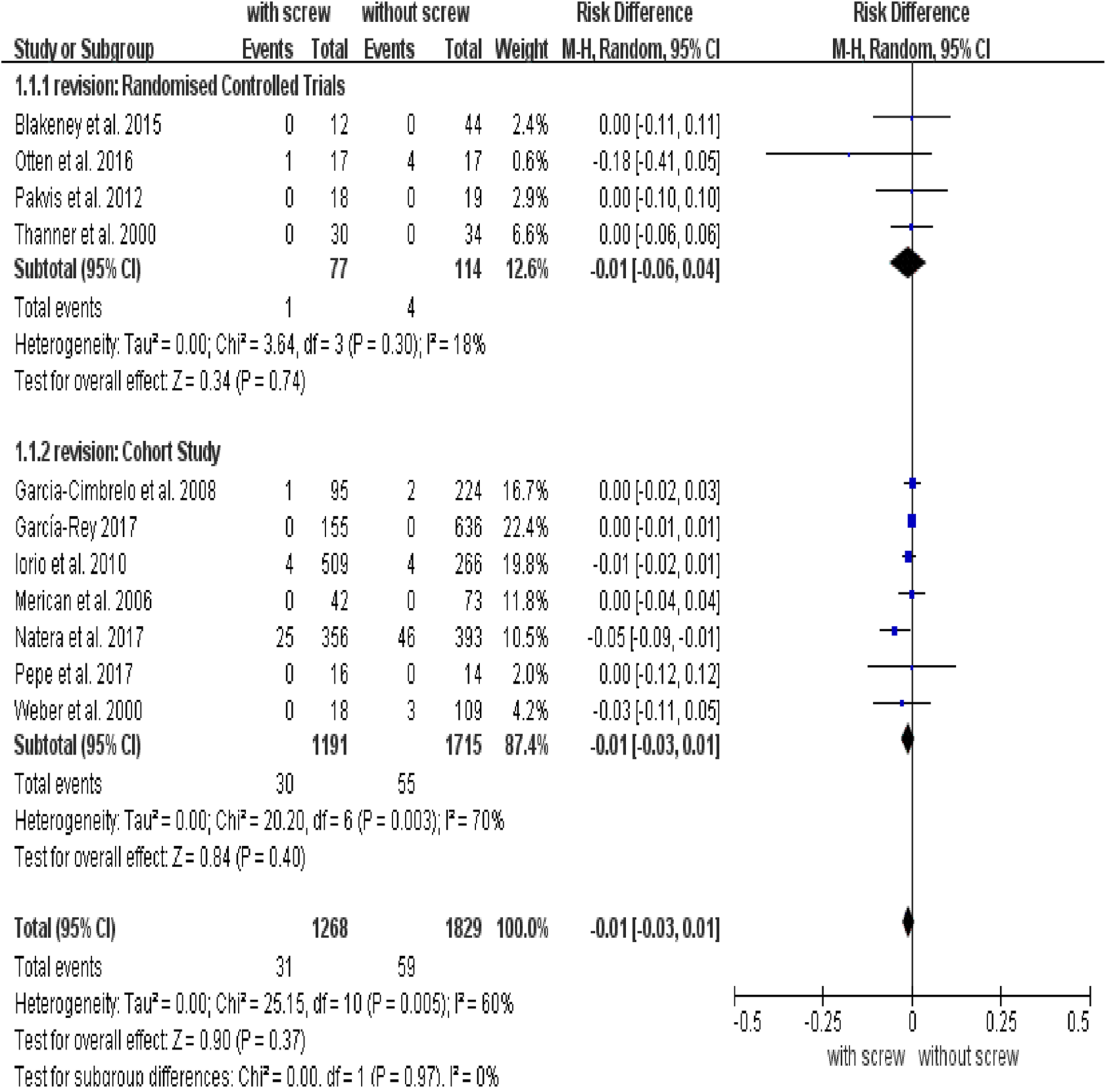
Forest plot of combined and subgroup analysis for the revision of acetabular cup

The analytic outcome of the revision used RD as effective measures, displaying that fixation with screw did not decrease the rate of revision (Fig. 4). The RD of the revision rate was − 0.01 (95% CI: − 0.03 to 0.01; *P* = 0.37). There were no conflicts between subgroups in revision (Fig. 4).

### Wear of prosthesis

**Fig. 5:**
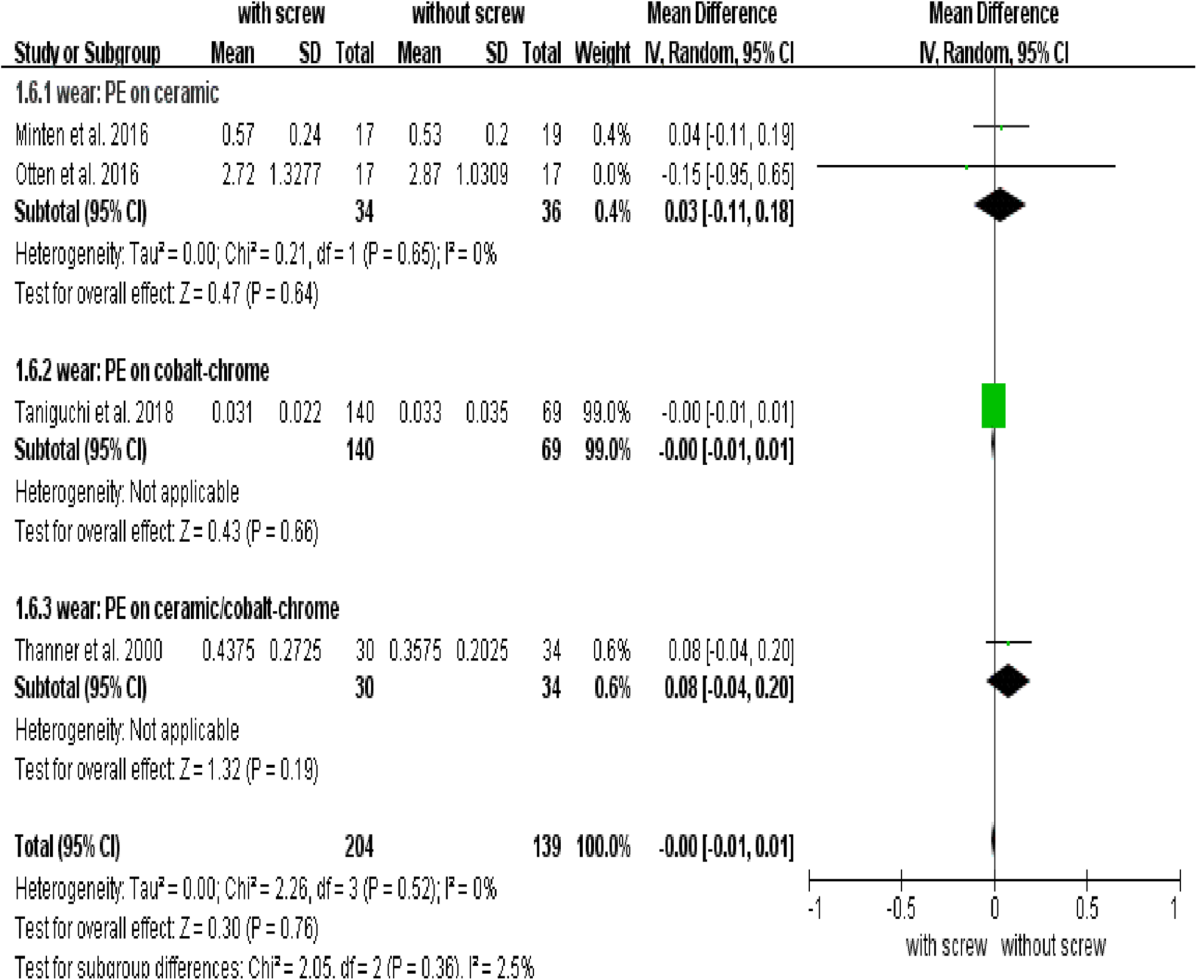
Forest plot of combined and subgroup analysis for the wear rate

There were no significant differences in the rate of wear between groups with and without screws (Fig. 5), the MD of wear was − 0.00 (95% CI: − 0.01 to 0.01; *P* = 0.76). There were no conflicts between subgroups of different bearing surfaces materials in wear (Fig. 5).

### Pain score and Harris hip score

**Fig. 6:**
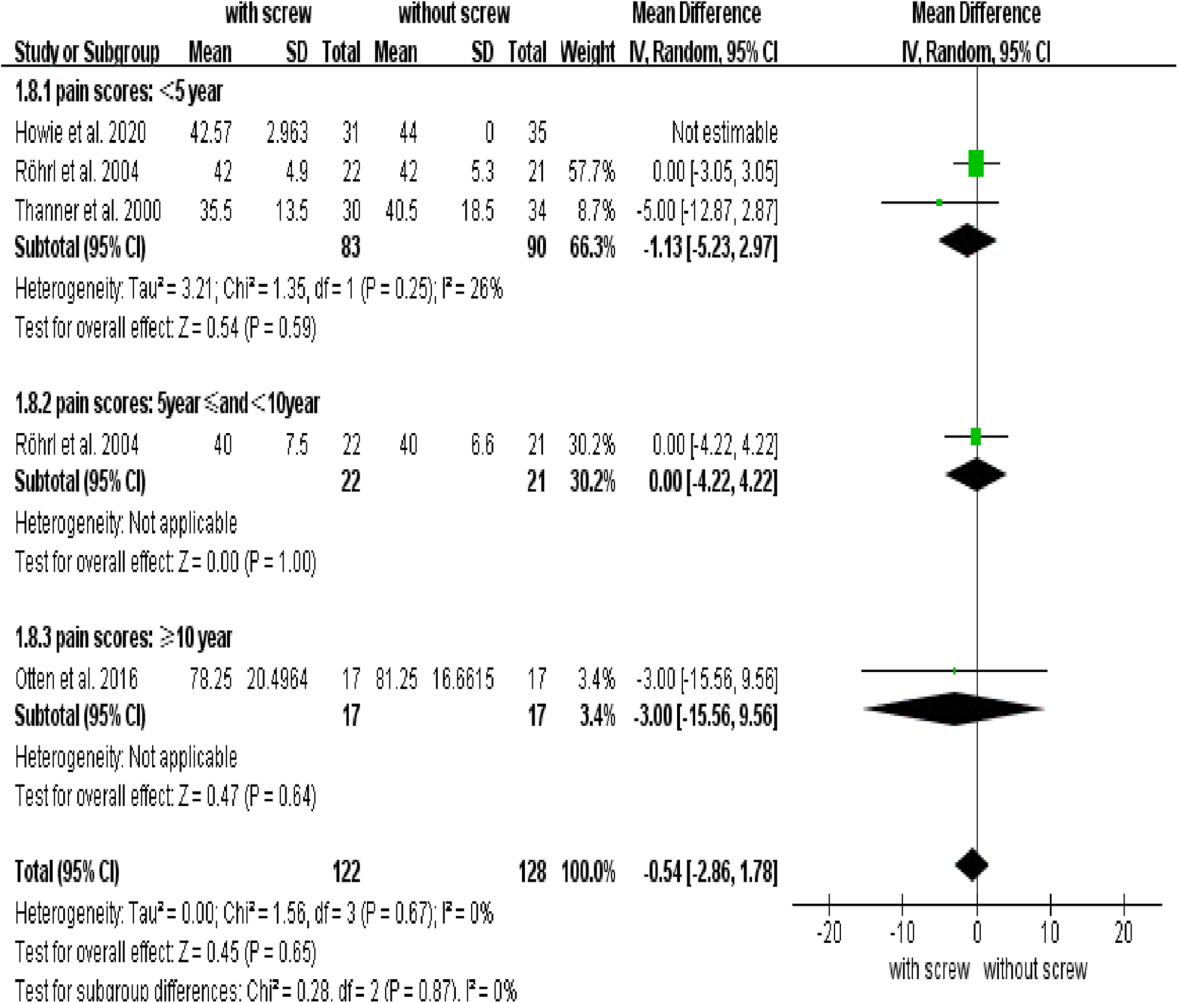
Forest plot of combined and subgroup analysis for pain score

**Fig. 7:**
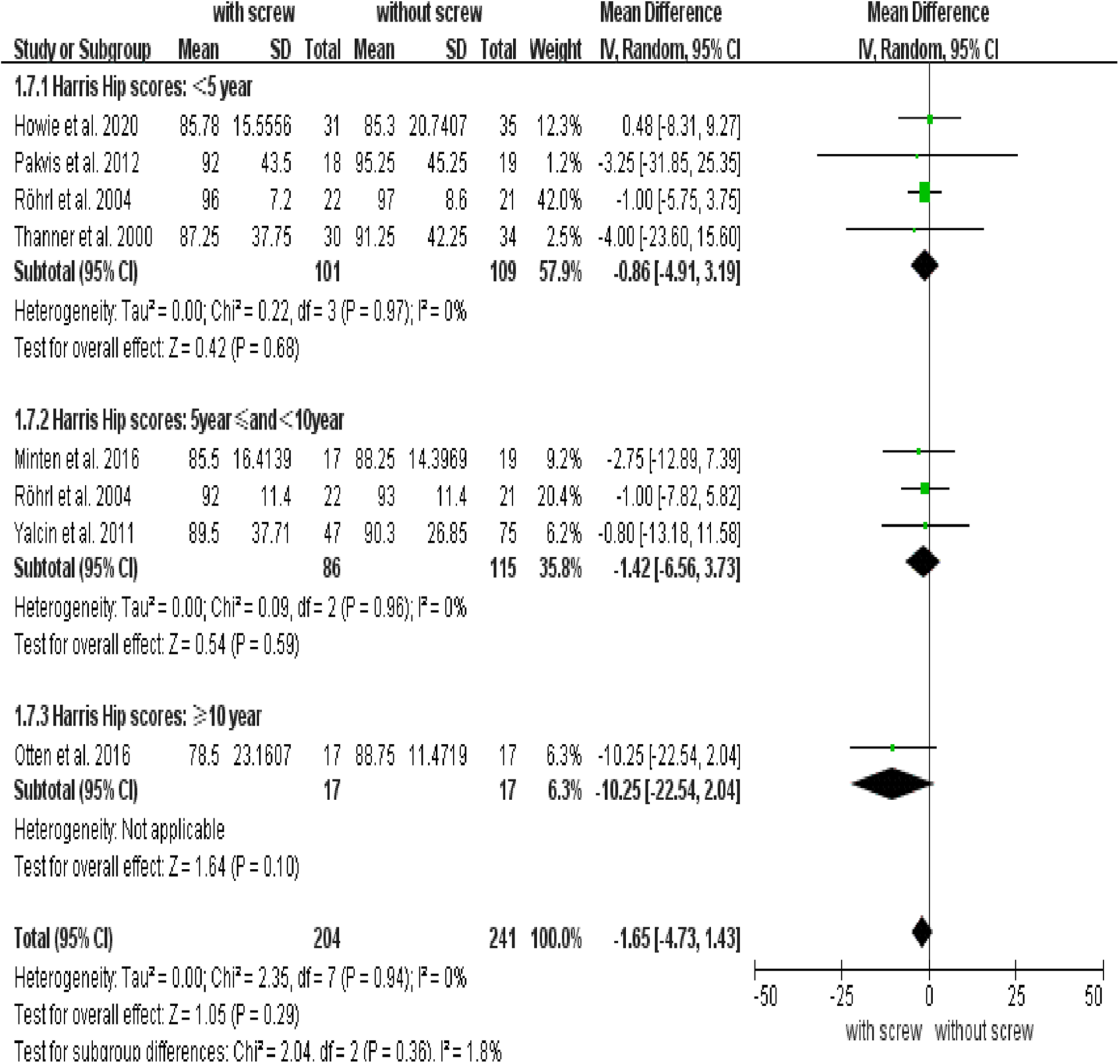
Forest plot of combined and subgroup analysis for Harris hip score

Our analysis revealed no differences in pain scores between groups (95% CI: − 2.86 to 1.78; *P* = 0.65; MD = − 0.54) (Fig. 6), and there was no difference in HHS between groups (95% CI: − 4.73 and 1.43; *P* = 0.29; MD = − 1.65) (Fig. 7). There were no conflicts between subgroups in pain scores and HHS (Figs. 6, 7).

### Risk of bias across studies

**Fig. 8:**
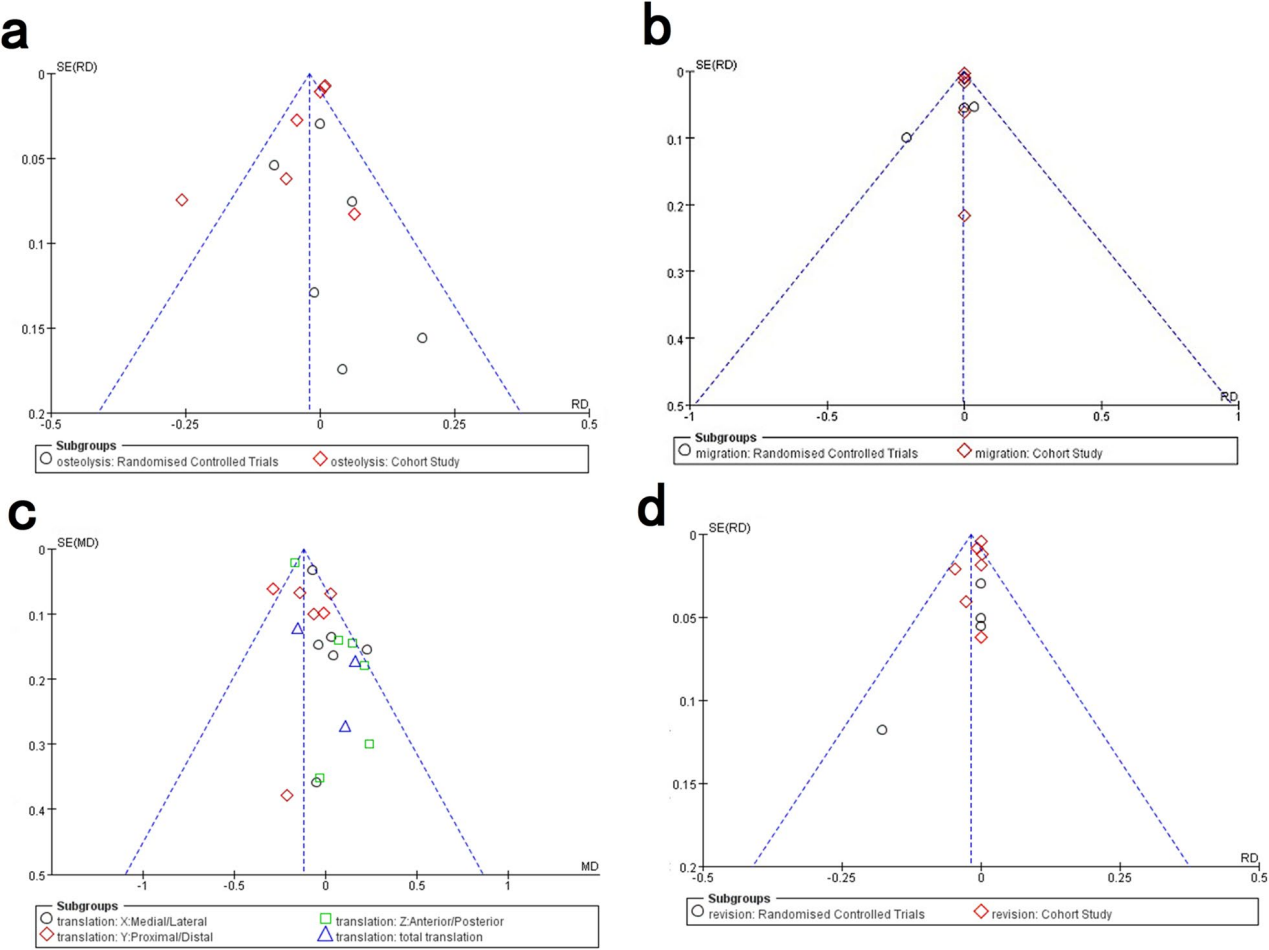
Funnel plot of publication bias for studies with the outcomes: **a** osteolysis, **b** migration, **c** translation, and **d** revision

Publication bias has long been recognized as a problem. In this review, we investigated publication bias by the funnel plots for the groups that included more than ten studies (Fig. 8). There was some publication bias among the included studies as well as inevitable clinical heterogeneity between the included studies. The differences were found in relation to study type, operative approach, acetabular component, and follow-up time.

### Sensitivity analysis

We conducted a special subgroup analysis for RCTs in the outcomes of osteolysis, migration, and revision, and found no difference between RCTs subgroup analysis and pooled analysis. There was one non-RCT in the analysis of wear, and the comprehensive analysis result did not change after this study was removed. There was also only one non-RCT in the analysis of HHS, and the result did not change after this study was removed. All the studies included in the outcomes of pain, translation, and rotation were RCTs. We made a sensitivity analysis by using the “available case analysis” instead of “ITT analysis using imputation” for all outcomes, and the results are described in Table 3. When changing the analysis method, the results showed no change in osteolysis, migration, rotation, revision, wear, pain scores, or HHS after the sensitivity analysis. The pooled analysis result of a translation by available case analysis was different from that by ITT analysis using imputation, indicating that cups without screws showed more translation (95% CI: − 0.13 to − 0.01; *P* = 0.03; MD = − 0.07). Interestingly, there was no significant difference in the subgroup analysis including *x*-axis (95% CI: − 0.12 to 0.02; *P* = 0.13; MD = − 0.05), *y*-axis (95% CI: − 0.23 to 0.00; *P* = 0.06; MD = − 0.11), *z*-axis (95% CI: − 0.17 to 0.21; *P* = 0.85; MD = 0.02), and total translation (95% CI: − 0.23 to 0.20; *P* = 0.87; MD = − 0.02). We also conducted a sensitivity analysis by using relative effect measures (RR) instead of absolute measures (RD) as an effective measure for osteolysis, migration, and revision (Table 4). There were no changes in the osteolysis and migration results, while the revision results changed. The pooled analysis showed that the risk of revision was higher in the group without screws (95% CI: 0.39–0.89; *P* = 0.01; RR = 0.59). A significant difference in revision was observed in the subgroup of cohort studies (95% CI: 0.40–0.94; *P* = 0.02; RR = 0.61), but there was still no difference between groups in the subgroup of RCTs (95% CI: 0.03–2.01; *P* = 0.19; RR = 0.25).

**Table 3:** The sensitivity analysis using the “available case analysis”

Outcomes	Studies (*n*)	THAs (*n*)	Effective measures	RD/MD (95% CI)	*P*-value	Heterogeneity	
						*I*^2^ (%)	*P*_*h*_
Osteolysis	13	3083	RD	− 0.01 (− 0.04, 0.02)	0.34	74	< 0.00001
Migration	10	2392	RD	0.00 (− 0.00, 0.00)	0.98	0	0.73
Translation	6	845	MD	− 0.07 (− 0.13, − 0.01)	0.03	38	0.04
Rotation	4	480	MD	− 0.32 (− 0.82, 0.19)	0.22	83	< 0.00001
Revision	11	3067	RD	− 0.01 (− 0.02, 0.01)	0.38	57	0.01
Wear	4	296	MD	− 0.00 (− 0.01, 0.01)	0.73	0	0.69
Pain scores	5	224	MD	− 0.51 (− 2.88, 1.86)	0.67	0	0.69
HHS	8	409	MD	− 1.41 (− 4.63, 1.81)	0.39	0	0.98

**Table 4:** The sensitivity analysis using RR as effective measures

Outcomes and subgroup analysis	Studies (*n*)	THAs (*n*)	Effective measures	RR (95% CI)	*P*-value	Heterogeneity	
						*I*^2^ (%)	*P*_*h*_
Osteolysis	13	3130	RR	0.92 (0.59, 1.43)	0.71	32	0.15
RCT	6	295	RR	1.30 (0.72, 2.34)	0.38	0	0.50
Cohort study	7	2835	RR	0.80 (0.42, 1.49)	0.47	41	0.13
Migration	10	2392	RR	0.57 (0.03, 11.17)	0.71	61	0.11
RCT	3	139	RR	0.57 (0.03, 11.17)	0.71	61	0.11
Cohort study	7	2253	RR	NA	NA	NA	NA
Revision	11	3097	RR	0.59 (0.39, 0.89)	0.01	0	0.90
RCT	4	188	RR	0.25 (0.03, 2.01)	0.19	NA	NA
Cohort study	7	2906	RR	0.61 (0.40, 0.94)	0.02	0	0.94

## Discussion

Total hip arthroplasty is commonly performed all over the world [32]. Most acetabular prostheses have screw holes reserved for adding screws. Some surgeons believe that adding screws, even routinely, can increase the stability of the prosthesis [2, 33]. However, some surgeons believe that satisfactory results can be achieved without adding screws and that adding screws can lead to some additional complications [4, 34]. Still, as there are no definitive guidelines, it remains controversial whether screws should be added. This review showed that additional screws did not lead to clinically important improvement in the stability of acetabular cups. There was no difference in revision, HHS, or pain scores between the groups with and without screws.

The stability of the acetabular cup is an important factor affecting the success of the surgery. Osteolysis is the main cause of aseptic loosening of the acetabular prosthesis. There are many reasons for osteolysis, the most important being particle disease [35]. Previous studies have shown that wear particles could enter screw holes and induce granulomas that are composed of many macrophages laden with wear particles [1]. Cytokines produced by particle-stimulated macrophages interfere with the osseointegration and cause osteolysis [35, 36]. A magnetic resonance imaging study has shown that obvious osteolysis occurred around acetabular screws [3]. However, some studies suggested that osteolysis is not necessarily related to screws [23, 37]. In our study, eight RCTs and seven cohort studies about osteolysis were included. After summary analysis, our results showed that additional screw fixation did not increase the incidence of osteolysis.

The initial stability of the cementless acetabular cup mainly depends on the press-fit, while the later stability mainly depends on the bone ingrowth. The addition of screws is designed to enhance the stability of the acetabular prosthesis. Most studies have suggested that the addition of screws can increase the initial stability of the acetabular cups [38]. Yet an in vitro study showed that satisfactory stability could be achieved through simple press-fit without screw use, and the addition of screws was only needed when the bone condition was poor [39]. Our results showed that press-fit with additional screw fixation did not reduce clinically relevant cup loosening or migration. Moreover, our study showed no statistical difference between cups with or without screw fixation in translation and rotation. Subgroup analysis for rotation of acetabular cup showed that the rotation degree at the *x*-axis and *z*-axis was greater in the group without screws, and there was no difference between groups in the rotation degree at *y*-axis. Usually, the orientation of screws was roughly the same as the *y*-axis and perpendicular to the *x*-axis and *z*-axis. This may explain why the screws limited the rotation of the cup in the *x*-axis and *z*-axis better than the *y*-axis; further research is needed to confirm this. The translation of cups was greater in the non-screw group compared with the screw group in our sensitivity analysis. Nevertheless, there was no difference in translation between the screw cups and the non-screw cups in the subgroups of *x*-axis, *y*-axis, *z*-axis, and total translation. Our sensitivity analysis still showed no difference in rotation and clinically relevant migration between the two groups.

We analyzed the effect of fixation with and without screws on the revision rate of the acetabular cup. When we used RD as an effect measure, there was no significant difference in acetabular revision between the groups with and without screws. However, in our sensitivity analysis, when RR was used as an effect measure, the subgroup analysis conclusion of the RCTs was in conflict with that of the cohort studies. The relative effect measures (RR) were more consistent than absolute measures (RD) [20]. Our meta-analysis included some trials with zero events in both treatment and control groups. Studies have shown that RR and RD effect measures produce different results in zero total event trials, and that the results of RR are more conservative [40]. Subgroup results of RCTs still showed no association between screws and revision rate, while subgroup results of cohort studies showed that additional screw fixation reduced revision rates. We evaluated the included cohort studies and found that the study bias was relatively large. One of the studies was a multicenter study involving multiple acetabular cups, multiple surgical approaches, and multiple surgeons, which significantly influenced the conclusions of the overall pooled analysis [10]. Therefore, the subgroup conclusion of the cohort studies in revision was less reliable and should be taken with caution. The subgroup conclusion of the RCTs was recommended.

Studies have shown that wear particles are important in osteolysis [35, 41]. After particles are produced by wear, macrophages are induced to migrate to local site of particles. Then a variety of inflammatory factors are released from macrophages, causing osteoclast activation and osteoblast inhibition [35, 42]. It has been suggested that changes in cup alignment after screw fixation might affect the wear of acetabulum components [43]. Our results showed that there was no difference in wear rate between the acetabular component with and without screws. Previous studies have shown that the wear of polyethylene liner was more serious than ceramic liner, and the effect on osteolysis of polyethylene particles was more serious than ceramic particles [35, 44, 45]. All the four studies included in the wear analysis used polyethylene as liner [9, 11, 19, 28], so the bias caused by different liners was excluded. The femoral head materials used in the four studies are different, including ceramic and cobalt-chrome. Therefore, we conducted a subgroup analysis for different materials. In three studies [9, 11, 19], the groups with and without screws used the same head materials, while in one study, the groups with and without screws used unequal femoral head materials. Our study confirmed that there was no difference in wear rate between the two groups when the liner and femoral head materials were used in the same way, indicating that the presence of screws had no effect on wear rate.

We also analyzed the HHS and pain score, which are closely related to the postoperative life quality of patients. Our results revealed no significant difference in pain scores and HHS between the groups with and without screws.

The use of screws to fix the cementless acetabular cup during THA has been controversial and widely discussed. Over recent years, a steady stream of research comparing the acetabular cup with and without screws has been published. This systematic review includes the latest studies comparing cementless acetabular cups with or without screws in THA through a search of a variety of databases, and provides sufficient evidence-based medical evidence.

The present study still has some limitations. The first limitation is related to the publication bias of the included studies. We only tested for publication bias of four analyses by funnel plot and did not further test by Egger’s or Begg’s test. Second, there is heterogeneity in the included studies: the studies differed in relation to study type, operative approach, acetabular component, and follow-up time. Accordingly, subgroup analysis and sensitivity analysis were performed to reduce heterogeneity and confirm the results.

## Conclusion

This study shows that additional screw fixation does not increase stability. Generally, press-fit can achieve good acetabular cup stability and there is no need to add screws. Our analysis showed no difference in osteolysis and clinically relevant migration of acetabular cups between groups with and without screws. Moreover, our pooled data showed no association between screws and revision rate. There was no difference in wear between the groups with and without screws. In addition, our study revealed no differences in pain scores and HHS between hips with and without screws use. Therefore, these findings suggest that additional screws might not be required for press-fit fixation of cementless acetabular cups.

## Data Availability

All data and materials can be retrieved from the references and articles included in the systematic review.

## Abbreviations

THA: Total hip arthroplasty
HHS: Harris hip scores
RCTs: Randomized controlled trials
NOS: Newcastle–Ottawa scale
RD: Risk difference
CI: Confidence interval
MD: Mean difference
RR: Risk ratio
NA: Not available
Ti: Titanium
HA: Hydroxyapatite
PE: Polyethylene
UHMW: Ultrahigh molecular weight
